# Emerging Anti-Mitotic Activities and Other Bioactivities of Sesquiterpene Compounds upon Human Cells

**DOI:** 10.3390/molecules22030459

**Published:** 2017-03-13

**Authors:** Alessandra Bosco, Roy M. Golsteyn

**Affiliations:** Natural Product and Cancer Cell Laboratories, Department of Biological Sciences, 4401 University Dr, University of Lethbridge, Lethbridge, AB T1K 3M4, Canada; alessandra.bosco@uleth.ca

**Keywords:** 6-*O*-Angeloylplenolin, α-methylene-γ-lactone, Asteraceae, checkpoint adaptation, coronopilin, *Gaillardia aristata*, oxozoapatlin, psilostachyins A and C, sesquiterpene lactone

## Abstract

We review the bio-activities of natural product sesquiterpenes and present the first description of their effects upon mitosis. This type of biological effect upon cells is unexpected because sesquiterpenes are believed to inactivate proteins through Michael-type additions that cause non-specific cytotoxicity. Yet, certain types of sesquiterpenes can arrest cells in mitosis as measured by cell biology, biochemical and imaging techniques. We have listed the sesquiterpenes that arrest cells in mitosis and analyzed the biological data that support those observations. In view of the biochemical complexity of mitosis, we propose that a subset of sesquiterpenes have a unique chemical structure that can target a precise protein(s) required for mitosis. Since the process of mitotic arrest precedes that of cell death, it is possible that some sesquiterpenes that are currently classified as cytotoxic might also induce a mitotic arrest. Our analysis provides a new perspective of sesquiterpene chemical biology.

## 1. Introduction

**Figure 1:**
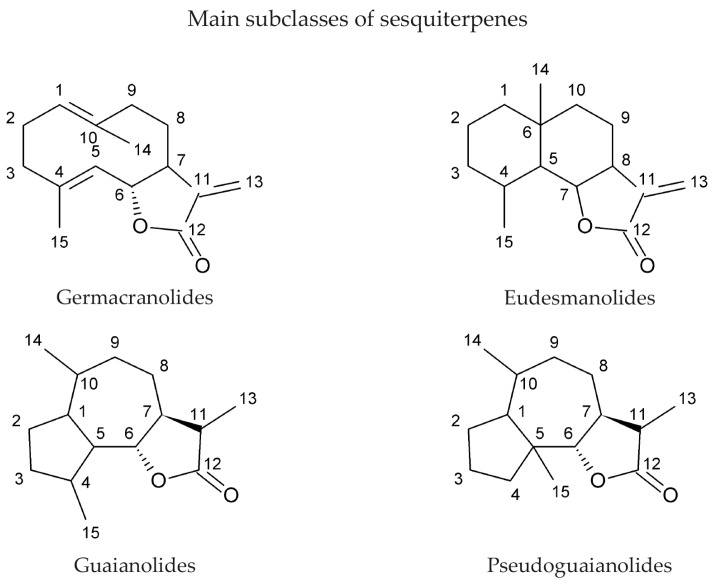
The chemical structures representing the main subclasses of sesquiterpenes are shown. The positions of carbons are numbered.

Sesquiterpene lactones are a class of natural product chemicals that are commonly synthesized by plant species. They are colourless, stable, and lipophilic chemicals that have a 15-carbon core structure (hence the prefix sesqui-), which is derived from the synthesis of three isoprene units and a five-member lactone ring [1,2]. Lactone rings are cyclic hydroxycarboxylic acid esters. Sesquiterpenes are classified into different subgroups based on their chemical structure (Figure 1); the largest subgroup is the germacranolides, which contain a 10-membered ring, whereas eudesmanolides are bicyclic 6/6 compounds, and guaianolides and pseudoguaianolides are bicyclic 5/7 compounds [3,4]. Sesquiterpenes can be acyclic, although the vast majority of them are cyclic and contain a lactone ring. The lactone component is characterized by an α-methylene-γ-lactone structure, which is an oxygen-containing ring with a carbonyl moiety at the β position (O=C‒C=CH_2_) [2,5] (Figure 1). The lactone is thought to be responsible for the majority of biological activities induced by sesquiterpene lactones when administered to organisms or cells. The unsaturated carbonyl structures react by a Michael-type addition with nucleophiles in biological systems, such as the sulfhydryl group in the amino acid cysteine [6,7,8]. One might assume that alkylation of thiol groups would have a non-specific effect upon cells because over 90% of the polypeptides encoded by the human genome contain a cysteine amino acid [9]. By testing sesquiterpene lactones in cell-based assays, however, it has been found that some have very specific effects, and possibly specific targets in cells. We review the literature of the bio-activity of sesquiterpene lactones and describe that a small number of them arrest cells in mitosis.

## 2. Sesquiterpene Lactones as Natural Products and Their Sources

Over 5000 sesquiterpenes have been identified from plant sources [5,10]. Sesquiterpenes are commonly found in a number of plant taxonomical families (Table 1). The Asteraceae family is particularly rich in sesquiterpene lactones, with over 3000 reported structures [10,11]. These chemicals can account up to 3% of the dry weight of species as in the case of tenulin, produced by *Helenium amarum* [12]. Some plant species from Asteraceae have the ability to shift the production of classes of terpenoids in response to herbivory, and store compounds in tissues upon which herbivores feed, such as leaves, trichomes, phyllaries or achenes [13]. In fact, some sesquiterpenes function as a deterrent to grazing by sheep and cattle, and are toxic to various insects [14,15,16,17]. The large number of structurally distinct sesquiterpene lactones in plants is directed by many sesquiterpene synthases encoded by the genome of a species. Chemical structure diversity across species correlates with the genetic diversity of synthases across species [18,19,20]. In addition, abiotic chemical modifications, such as thermolysis, can give rise to new structures [5,14,21,22]. For example, the two recombinant sesquiterpene synthases prepared from *Abies grandis* (grand fir), δ-selinene synthase and γ-humulene synthase, can produce more than 30 sesquiterpene olefins each using the acyclic precursor farnesyl diphosphate [21]. From the representative classes of sesquiterpene lactones, germacranolides are derived from the oxidation of the 3 carbon side chain, which results in the lactone ring, and eudesmanolides, guaianolides and pseudoguaianolides are derived from germacranolides [13].

## 3. Biological Activities of Sesquiterpene Lactones

As a consequence of sesquiterpene lactone structural diversity, these chemicals have a range of effects on the physiology of metazoan species, including humans. These compounds were also reported to have antifungal and antibacterial activities, which are described elsewhere [23,24,25,26,27,28].

### 3.1. Effects upon Insects and Grazing Herbivores

Sesquiterpene lactones play important roles in the defense of the plants against herbivores such as insects or mammals. These compounds repel or poison grazing herbivores and attract parasite predators that attack the herbivore organism, thereby decreasing feeding upon the producer plants. The anti-feedant properties of sesquiterpene lactones were first demonstrated by Burnett et al. in 1974, who conducted larval feeding experiments on three *Vernonia* spp. [29]. They reported that two sesquiterpene lactones, glaucolide A and alantolactone, deterred feeding and reduced the survival of several insect species [29]. In particular, a concentration of glaucolide A lower than 0.5% in *Vernonia* spp. results in increased levels of feeding, whereas concentrations >1.0% of glaucolide A reduced feeding levels. This observation was supported by Rossiter et al. who observed that *Helianthus* spp. deterred feeding by sunflower moth larvae by 50% when the plants contained more than 1% dry weight of the sesquiterpene 8β-sarracinoyloxycumambranolide (8β-SC) [30]. Many other studies highlight the importance of sesquiterpenes in defending the plant from insects [15,16,17,20,24,31,32]. Volatile sesquiterpenes can repel or attract insects; citrus leaves release higher amounts of sesquiterpenes when in a juvenile state than a mature state [33]. Volatile sesquiterpenes are released to attract parasite predators, which help the plant defend itself against insect herbivores [34].

Mammals are also affected by contact with sesquiterpene lactones, either as a consequence of their toxicity or, in some cases, by taste. The observation that mammals respond adversely to sesquiterpene lactones suggests that the capacity to produce these secondary metabolites may have coevolved with grazing mammals [35]. For instance, rabbits and deer show avoidance behavior to the sesquiterpene glaucolide A from *Vernonia* spp. [35]. In addition, *Helenium amarum*, produces the sesquiterpene, tenulin, which is toxic to livestock [12]. The sesquiterpene lactone, helenalin, isolated from *Helenium microcephalum* is toxic to cattle, sheep and goats [36]. Overall, there is a considerable number of sesquiterpene lactones, many of which were isolated from Asteraceae species, which are reported to affect the survival of mammals or have mammalian feeding deterrent properties [24].

### 3.2. Effects of Sesquiterpene Lactones upon Humans

The chemical reactivity of sesquiterpene lactones and their effects upon grazing mammals make it likely that these molecules would affect human physiology. More than 200 species of Asteraceae have been reported to cause contact dermatitis, with cases documented in Australia, Europe and America [33,37,38,39]. This condition is due to an inflammation of the skin after direct contact with plants [37]. It consists of localized itchy and burning rashes on skin that in some cases develop blisters. The Asteraceae plants in particular cause a more widespread eczema due to contact with airborne particles of the plant, defining the Compositae (a synonym of the Asteraceae) dermatitis [39], and sesquiterpene lactones have been identified as the causative agent [37,40,41,42]. The methylene group attached to the lactone ring is necessary but not sufficient to induce contact dermatitis [40]. Sesquiterpene lactones that are structurally different can cause cross-reactions, whereas identical sesquiterpenes from different plant species can cause false reactions. As a result of the great number of sesquiterpenes, the cross-reactions among them and the different proportions in plant species, the clinical description of contact dermatitis is complex [40,43].

### 3.3. Medicinal Properties of Sesquiterpene Lactones

Several medically important sesquiterpenes have been identified. They have been used for treatments of cardiovascular diseases [2,44], ulcers [45], or minor illnesses and symptoms such as diarrhoea, flu, neurodegeneration, migraines, burns, and pain [10,46,47,48]. *Ambrosia tenuifolia* is an Asteraceae plant that harbours psilostachyins, which are sesquiterpenes with anti-parasitic activity [24,49]. These compounds are active against *Leishmania* spp., which are responsible for severe forms of leishmaniasis, with an IC_50_ value of 0.12 µg/mL [50].

Artemisinin is one of the most significant medicines at a global level. It is a sesquiterpene discovered and isolated from the Chinese herb *Artemisia annua* by Tu YouYou [51,52]. For this discovery Tu YouYou was awarded the 2015 Nobel Prize for Medicine [53]. Artemisinin is active against *Plasmodium falciparum*, the causative organism of malaria. A derivative of artemisinin is now a standard worldwide treatment for malaria [52,54,55,56,57]. Although the mechanism of action of artemisinin is not well understood, studies suggest that its endoperoxide bridge generates free radicals that damage vital proteins in the parasite system, resulting in its death [58,59,60]. It was shown that artemisinin generates toxic free radicals, which interact with the intraparasitic heme of the parasite [61].

### 3.4. Anti-Inflammatory Effects

Sesquiterpene lactones modulate several inflammatory processes, such as oxidative phosphorylation, platelet aggregation, histamine and serotonin release. However, the main inflammatory response inhibited by sesquiterpenes involves Nuclear Factor-kappa B (NF-κB) [62,63]. A comprehensive study by Bork et al. showed that 54 Mexican Indian medicinal plants, all rich in sesquiterpene lactones, had potent inhibitory effects on the NF-κB pathway [64]. NF-κB is a family of proteins that control DNA transcription, cytokine production, and cell survival. The proteins form either a hetero- or homo-dimer cytoplasmic complex comprised of the subunits, p50 and p65. Its activity is tightly regulated by interaction with the natural inhibitor IκB, which sequesters the NF-κB dimer in the cytosol [65]. Pathogenic or inflammatory stimuli lead to the production of reactive oxygen species (ROS) and phosphorylation and ubiquitination of IκB. Once IκB is ubiquitinated, it is recognized by the proteasome and degraded [66,67]. The absence of IκB leaves the NF-κB dimer free to translocate to the nucleus and induce transcription of target genes [68]. NF-κB regulates over 150 genes in pathways that mediate inflammatory or immune processes in response to injury, or bacterial and viral infections [62]. As elucidated by Rüngeler et al., a possible mechanism of inhibition of NF-κB by sesquiterpenes is through alkylation of the amino acids Cys38 and Cys120 in the DNA-binding domain of the p65 subunit [62]. Cys38 forms a hydrogen bond with the backbone of the κB-DNA motif, participating in DNA binding. The sulfur atom of Cys120 is in proximity to Cys38, and the space between the two amino acids normally positions the phenol ring of Tyr36, which is essential for DNA binding. A sesquiterpene lactone adduct in which both sulfur atoms are alkylated creates a cross link between Cys38 and Cys120 in the p65 subunit, and impairs DNA binding.

A comprehensive study conducted by Siedle et al. characterized 103 sesquiterpene lactones from 6 subclasses in their capacity to inhibit NF-κB DNA binding [63]. They found that the majority of active sesquiterpene lactones belonged to the guaianolides subclass and that the presence of the α,β-unsaturated carbonyl group played a major role in cytotoxicity instead of the α-methylene-γ-lactone groups [63]. Zerumbone and parthenolide were two of the sesquiterpenes with anti-inflammatory activities [69,70]. Zerumbone treatment at 50 µM for 12 h inhibited the activation of NF-κB and NF-κB-regulated gene expression induced by carcinogens and various inflammatory agents (such as okadaic acid, tumour necrosis factor (TNF), cigarette smoke and hydrogen peroxide) on H1299 lung adenocarcinoma, KBM-5 chronic myelogenous leukemia, A293 embryonic kidney, and FaDu squamous cell carcinoma cell lines. Zerumbone treatment at 25 µM for 12 h also reduced expression of NF-κB-dependent gene products involved in cell proliferation, anti-apoptosis and invasion [69]. Parthenolide, a sesquiterpene lactone that is structurally different from zerumbone, induced apoptosis in human acute myelogenous leukemia stem and progenitor cells through inhibition of NF-κB, proapoptotic activation of p53, and increasing amounts of ROS [70]. Parthenolide was also described as an inhibitor of NF-κB activation in HeLa cells by binding directly to IκB-kinase (IKK), which prevents it from phosphorylating the IκB [71] and maintaining IκB association with NF-κB.

### 3.5. Anti-Tumour Activities

There are numerous reports describing the activities of sesquiterpenoids upon different pathways in human cancer cells [3,72] of which the predominant effect is cytotoxicity. Lee et al. investigated the cytotoxicity of sesquiterpenes on normal lung fibroblastic cells WI-38, HEp2 epidermoid carcinoma of larynx cells and the W18-Va2 buccal mucosa (fibroblast-like) cells and reported that 16 out of 18 sesquiterpenoids showed cytotoxicity to three cell lines tested, the most active being helenalin with an IC_50_ ranging from 0.03 to 0.18 μg/mL [73]. It was later postulated that helenalin reacts with the cysteines of telomerase proteins and inactivates enzyme activity in T-cell leukemia (Jurkat cells) and HL60 promyelocytic leukemia [74]. Telomerase maintains telomeres length ensuring immortality of cancer cells. Lee et al. concluded that the unsaturated carbonyl (O=C‒C=CH_2_), independently of whether it was included in a lactone or cyclopentanone, was required for cytotoxicity [2,73]. However, the presence of additional alkylating groups such as cyclopentanone, or α-methylene-γ-lactone appeared to enhance cytotoxicity, as the latter plays an important role in alkylating enzymes [75,76,77]. Other structure-cytotoxicity studies on sesquiterpenoids showed that α-methylene-γ-lactones react rapidly with cysteine to form stable adducts, whereas endocyclic α,β-unsaturated-γ-lactones react slowly with cysteine, to form unstable adducts [6]. Furthermore, Lee and Hall established that the α-methylene-γ-lactone moiety, a β-unsaturated cyclopentanone ring or an α-epoxycyclopentanone system are the essential structures for anti-tumour activity in vivo [78,79]. Overall, sesquiterpene lactones selectively alkylate nucleophilic groups in many enzymes, including those involved in the control of cell division [2,24]. Kupchan suggested that the tumour inhibitory activity is selective for thiols, for sulfhydryl enzymes and for sulfhydryl groups within enzymes [80]. For example, phosphofructokinase and other sulfhydryl enzymes from rabbit skeletal muscle lose their activity in vitro after reaction with sesquiterpenes [81,82]. It was found that sesquiterpene lactones inhibited DNA polymerase and thymidylate synthase enzymatic activity in tumour cells, thereby inhibiting nuclear DNA synthesis [77,78,83].

Increases in NF-κB activity can contribute to cancer development and progression, and it provides a mechanism by which tumour cells escape immune surveillance and resist chemotherapy and radiotherapy [84]. This nuclear factor plays an important role in prevention of carcinogenesis, and many human tumours have a constitutively active NF-κB [85]. When NF-κB is active it promotes cell cycle entry and inhibits apoptosis [85]. Therefore, NF-κB inhibitors can sensitize tumour cells to apoptosis signaling pathways activated by multiple stimuli, or prevent cell proliferation, or to the effects of other anti-tumour agents.

### 3.6. Clinical Trials of Sesquiterpene Lactones

Three sesquiterpene lactones entered clinical trials by virtue of the chemical properties such as alkylating center reactivity, lipophilicity, molecular geometry and electronic features [72]. These compounds are: L12ADT, a peptide prodrug derived from thapsigargin isolated from *Thapsia*; artesunate, a derivative of artemisinin from *Artemisia annua* L. and dimethylamino-parthenolide (or LC-1), an analogue of parthenolide from *Tanacetum parthenum* (Table 2). These compounds have selective activities toward tumour and cancer stem cells by targeting specific signaling pathways involved in cell differentiation, cell proliferation, and apoptosis through mitochondrial and caspase signaling pathways and through an increase of the cytosolic concentration of calcium [72]. Artesunate has shown promising results in the treatment of laryngeal carcinomas, uveal melanomas and pituitary macroadenomas, and is currently undergoing phase I clinical trials against cervical intraepithelial neoplasia, colorectal cancer and other solid tumours [86,87,88,89,90]. Artesunate targets the iron group content by catalyzing the generation of free radicals from the bridged endoperoxide group [91]. Furthermore, artesunate reverses multi-drug resistance by reducing the adenosine triphosphate (ATP)-binding cassette subfamily G member 2 (ABCG2), a multidrug transporter, expressed in esophageal cancer [86,91]. L12ADT (8-*O*-(12-{l-leucinoylamino}dodecanoyl)-8-*O*-debutanoyl-thapsigargin) underwent phase I clinical trials for treatment of refractory, advanced or metastatic solid tumours, and is currently undergoing phase II clinical trials for the treatment of glioblastoma [92,93]. By targeting the sarco/endoplasmic reticulum (ER) calcium ATPase (SERCA) pump, thapsigargin causes apoptosis [94,95]. Dimethylamino-parthenolide (LC-1), an oral bioavailable parthenolide analogue [96], was investigated in a phase I trial against acute myeloid leukemia (AML), acute lymphoblastic leukemia (ALL) and other blood and lymph node cancers in 2012 [72,86]. However, the phase I clinical trial was suspended after one year [97].

## 4. Anti-Mitotic Activities of Sesquiterpenes

Research from our laboratory led us to identify a novel sesquiterpene lactone from the Asteraceae family member *Gaillardia aristata*, using phenotypic assays to detect anti-mitotic compounds. The discovery of an anti-mitotic sesquiterpene lactone was surprising to us because very few have been described to have this activity although more than 1500 publications have reported anti-cancer and anti-inflammatory properties [72,98,99]. Furthermore, the predicted mechanism of action of sesquiterpene lactones is counterintuitive to a protein target that would have a specific mitotic phenotype.

### 4.1. 6-*O*-Angeloylplenolin (6-OAP)

6-*O*-Angeloylplenolin (Table 3) was isolated from the Chinese medicinal herb, *Centipeda minima*, from the Asteraceae family [100]. 6-OAP has anti-proliferative properties on MM.1R, MM.1S, U266 and RPMI 8226 multiple myeloma cells and induces a G_2_/M-phase arrest. The arrest is characterized by an increase of cyclin B levels and a decrease in Tyr15 phosphorylatedcyclin dependent kinase 1 (Cdk1) [100]. Treatment of cells with 7.5 µM 6-OAP for 24 h induced a mitotic arrest, which in turn activated the spindle assembly checkpoint (SAC) proteins BubR1 and Mad2. The authors showed that 6-OAP facilitated the binding between the proteins Mad2 and Cdc20, which prevented the activation of the anaphase-promoting complex/cyclosome (APC/C). As a consequence, the levels of ubiquitinated cyclin B decreased, which is believed to prevent cyclin B degradation by the proteasome. The mitotic arrest induced by 6-OAP treatment was confirmed by observation of an increase of phosphorylated levels of histone H3 (PH3), and the formation of a mitotic spindle [100]. In a subsequent study, Liu et al. reported that 6-OAP inhibited the S-phase kinase-associated protein 1 (Skp1) in A549 lung adenocarcinoma and NCI-H1975 non-small cell lung cancer cell lines [33]. Skp1 is a component of the Skp1-Cullin-F-box containing (SCF) complex, an E3 ubiquitin ligase. This complex promotes the ubiquitination of regulatory proteins that targets them for degradation by the proteasome [101,102]. Computational docking analysis and co-immunoprecipitation analysis suggested that 6-OAP treatment at 7.5 μM binds Skp1 at the Skp1–Skp2 interface, and attenuates Skp1–Skp2 interaction. This causes dissociation and proteolysis of E3 ligase complexes nuclear interaction partner of Alk (NIPA), Skp2, and β-TRCP, and accumulation of their substrates cyclin B, p27 and E-cadherin. In a murine model in vivo, 20 mg/kg of 6-OAP for 30 days reduced tumour mass and prolonged mice survival. 6-OAP treatment in vivo did not affect body weight or serum concentration of control proteins. Another target of 6-OAP is the transcription factor named Signal Transducer and Activator of Transcription 3 (STAT3), which promotes STAT3-dependent Skp2 transcription [103]. Therefore, 6-OAP may repress Skp2 activity in a dual action by inhibition of STAT3 which impedes Skp2 transcription, and by binding to Skp1, causing dissociation and proteolysis of Skp2 [102,103].

### 4.2. 9β-Acetoxycostunolide and Santamarine

9β-Acetoxycostunolide (Table 3) is a derivative of the sesquiterpene lactone costunolide, and structurally related to santamarine (Table 3). Both compounds were isolated from the Asteraceae Chinese herb *Cyathocline purpurea*. Santamarine and 9β-acetoxycostunolide block L1210 murine leukemia cells in the G_2_/M phase of the cell cycle in a concentration- and time-dependent fashion with a corresponding decrease of cells in the G_1_ phase, and subsequent cell death. The duration of the treatments were 2 and 48 h, with concentrations of either compound ranging between 1 and 10 µM. However, the target causing the arrest had not been identified and it was not determined if cells were in mitosis [104].

### 4.3. Artemisinin and its Derivatives Artesunate and Dihydroartemisinin

Artemisinin is a sesquiterpene lactone isolated from the plant species *Artemisia annua* (Asteraceae). Its derivative, artesunate, is an effective therapy against malaria [52,53,56] (Table 3). Artesunate can induce a mitotic arrest in human cells in culture [105,106]. Artesunate induced a G_2_/Mphase arrest on four cell lines when applied at 26 μM for 48 h (J-Jhan and H69) or at 78 μM treatment for 24 h (HCT116 and U251) [105]. Although specific biochemical measures of mitosis were not described, further analysis showed that treated cells exhibited remote centrosomes and two nuclei per cell, indicating that the cells had duplicated the DNA but could not divide properly [105]. The multiple centrosomes, multiple spindles and multinucleated cells suggested that artesunate also caused a defect in cytokinesis. Dihydroartemisinin is another semi-synthetic derivative of artemisinin and was shown to have anti-cancer properties [106]. When HeLa cells were radiosensitized with 6 Gy X-radiation for 24 h and treated with 20 μM dihydroartemisinin, they exited the G_2_ block and downregulated the protein kinase Wee1, and upregulated cyclin B levels. Combined treatment with irradiation and dihydroartemisinin caused HeLa cells to abrogate the G_2_/M checkpoint and enter mitosis with a highly rearranged genome, eventually leading to mitotic cell death.

### 4.4. Coronopilin

Coronopilin (Table 3) is a sesquiterpene lactone isolated from *Ambrosia arborescens*, an aromatic plant that belongs to the Asteraceae family, and grows in western South America. Cotugno et al. found that treatment of the U937 leukemia cell line by 20 μM coronopilin for 24 h induced 52% of the cells to arrest in the G_2_/M phase, as compared to 12% of control samples [107]. The fraction of mitotic cells increased from 6.6% to 18.0% after treatment. The mitotic arrest was characterized by cyclin dependent kinase 1 (Cdk1) activity and sustained levels of cyclin B, and high levels of PH3. The authors found that coronopilin could bind covalently with tubulin and cause an increase of hyperpolymerized tubulin. The α,β-unsaturated carbonyl group of coronopilin is likely responsible for the activities of coronopilin because the structural analogue, dihydrocoronopilin, which lacked an α,β-unsaturated carbonyl group, did not inhibit leukemia cell population growth [107].

### 4.5. Costunolide

Costunolide (Table 3) is a sesquiterpene lactone isolated from *Michelia compressa*, a small tree that belongs to the Magnoliaceae family. Costunolide inhibits tubulin carboxypeptidase activity in Bt-549, MDA-MB-436 and MDA-MB-157 breast cancer cells in a manner similar to that of parthenolide (see parthenolide section), at concentrations ranging from 5 to 25 μM and at 6 h post treatment [108]. In HA22T/VGH hepatocellular carcinoma cells, treatment by costunolide (5 μM) caused a mitotic arrest as measured by an increase in PH3 positive cells from 3.6% in the control to 25.8% in treated cells [109]. Cells were arrested in metaphase as shown by the mitotic spindle and DNA configuration accompanied by upregulation of phosphorylated Cdc25c (Ser216) and cyclin B [109]. In a separate study, 100 nM costunolide at 24h post treatment had anti-proliferative activity upon MCF-7 breast cancer cells with low cytotoxicity, correlated with the interference with microtubule dynamics, forming short and dense microtubule fibers [110]. It was suggested that the interaction of costunolide with tubulin involves the nucleophilic reaction with sulfhydryl groups of cysteines [110,111]. However, the precise mechanism of the interaction between costunolide and tubulin was not shown experimentally. Rasul et al. also reported a G_2_/M-phase arrest by costunolide in T24 bladder cancer cells in which percentage of cells in G_2_/M increased from 13.8% in the control group to 25.6% or 41.3% in cells treated with 25 and 50 μM of costunolide for 24 h, respectively [112]. The precise mechanism of costunolide activity has not been characterized, although it seems to affect microtubule dynamics.

### 4.6. Dehydroleucodine

The sesquiterpene lactone dehydroleucodine (Table 3) was isolated from the aromatic herb *Artemisia douglasiana* (Asteraceae family). When applied to HeLa S3 cervix cancer cells for 24 h, 20 μM dehydroleucodine increased duration of the mitotic phase, which included an increase in the number of cells positive for PH3 [113]. The G_2_/M delay was also accompanied by decreased levels of cyclin B, in contrast to other results reviewed here. When treated cells exited mitosis, they did not undergo cell death but arrested in the G_1_ phase. However Costantino et al. found that dehydroleucodine induced DNA lesions after 8 h of treatment, characterized by presence of the phosphorylated protein kinase Ataxia-telangiectasia mutated (ATM), γ-H2AX, and increased levels of the DNA double-stranded breaks (DSBs) marker 53BP1. These findings suggest that DNA damage causes slower transitions through the S and G_2_/M phases followed by activation of apoptosis or senescence responses [113].

### 4.7. NP136

NP136 (Table 3) and the related compounds NP339 and NP176 are sesquiterpene lactones that were identified by a screen for natural products that modulate centriole number [114]. It was reported that these compounds do not affect the cell cycle when tested at 7.5 μM for 48 h. The compounds were tested at 7.5 ¼M, however, they reduced the number of centrosomal components of the mitotic spindle in mitotic HeLa cells. The effects of one of the compounds, NP339, was suggested to be linked to cysteine modification and the NF-κB pathway.

### 4.8. Parthenolide

The sesquiterpene lactone parthenolide was isolated from the feverfew, *Tanacetum parthenium* (Asteraceae family) (Table 3). Fonrose et al. showed that 10 μM parthenolide inhibited tubulin carboxypeptidase (TCP) in HeLa cells and impaired tumour progression [115]. Detyrosinated tubulin is a form of α-tubulin monomer that lacks the tyrosine at the C-terminus of the protein, which exposes a glutamic acid [116]. This form of detyrosinated tubulin destabilizes microtubules and impairs mitotic spindles, increasing the likelihood of tumour invasiveness and progression [116]. The removal of the C-terminal tyrosine residue is catalyzed by an ill-defined TCP, whereas the re-addition of tyrosine is mediated by the tubulin tyrosine ligase enzyme (TTL), which is frequently suppressed in tumour cells, leading to the accumulation of Glu-tubulin in these cells [117,118]. It is postulated that inhibition of TCP might reverse Glu-tubulin accumulation in tumour cells and restore normal tyr-tubulin levels. The α-methylene-γ-lactone moiety was indispensable for the inhibitory effect of parthenolide on TCP, which was not correlated to parthenolide inhibition of NF-κB pathway [115]. Parthenolide inhibited TCP in MDA-MB-157, MDA-MB-436, and Bt-549 breast carcinoma cells; it decreased the pool of detyrosinated tubulin without impairing the microtubule network [108]. As a consequence, parthenolide reduced microtentacle formation and tumour cell attachment. Tang et al. showed that 16 µM parthenolide inhibited U373 glioblastoma cells proliferation by causing a G_2_/M-phase arrest followed by apoptosis [119], but the mechanisms of its action were not elucidated and it was not reported if cells were in mitosis.

### 4.9. Psilostachyin A and Psilostachyin C

Psilostachyins A and C are sesquiterpene lactones isolated from the Asteraceae plant *Ambrosia artemisifolia* (Table 3). They decrease the percentage of MCF-7 breast cancer cells arrested in the G_2_ phase with a concomitant increase of mitotic cells, in an assay to detect compounds that relieve a DNA damage checkpoint [120]. Psilostachyin A and C (50 µM) induced a 40% and 50% increase in the number of cells to override G_2_ phase arrest, respectively. Cells that were not pretreated with a genotoxic agent (i.e., single treatments similar to those to detect other mitotic sesquiterpene lactones) also arrested in mitosis. Treated cells exhibited condensed chromosomes that failed to align, with mitotic spindles that were not properly organized. Microtubules in treated cells formed long and thick fibers, and no metaphase, anaphase or telophase arrangements were observed. Microtubule polymerization was not stimulated in vitro in presence of psilostachyins A and C, suggesting that these sesquiterpene lactones do not target microtubules or tubulin. Furthermore, to determine which reactive group was responsible for the activity, a mercapto-psilostachyin A derivative of the α-lactone was synthesized. This new compound no longer inhibited mitosis, demonstrating a requirement for the α,β-unsaturated carbonyl group for inhibitory activity. This is the first demonstration of a structure-activity relationship between the α,β-unsaturated carbonyl group and mitotic arrest.

### 4.10. α- and β-Santalols

α- And β-santalols are sesquiterpenes extracted from sandalwood oil, produced by the distillation of the heartwood of the *Santalum album* tree, of the Santalaceae family [121]. These compounds lack the lactone moiety (Table 3). Lee et al. found that these sesquiterpenes are toxic to seven different human head and neck squamous carcinomas cell lines (SCC-4, CAL 27, HSC-3, SCC-9, SCC-25, HN5, and HSC-2 HNSCC), and cause a G_2_/M-phase arrest at concentrations ranging from 7.7 to 45 µM, which corresponded to a maximal G_2_/M arrest [121]. Treated cells became rounded, arrested in mitosis and exhibited aberrant mitotic spindles (punctate, multipolar or monopolar spindles). These observations correlated with the ability of santalols to inhibit the polymerization of purified tubulin. In a turbidimetric assay, 50 µM α-santalol decreased polymerization of purified tubulin by 30% compared to 50 µM β-santalol. Rhodium protein docking simulation program lead to the prediction that both chemicals bind tubulin at the colchicine site, with a low affinity (K_i_ of 5.5 and 6.6 µM, respectively). The authors concluded that α- and β-santalols inhibit proliferation of several human head and neck squamous carcinomas with low potency, targeting tubulin polymerization and disrupting mitotic spindle formation [121].

Another study by Zhang et al. reported that 24 h treatment of human A431 epidermoid carcinoma and UACC-62 melanoma cells with α-santalol at concentrations above 50 µM reduced cell viability by 20%–30% and caused progressive accumulation of cells in G_2_/M-phase [122]. An increase of expression of cyclin B occurred in the epidermoid carcinoma, whereas a downregulation of cyclin B and A in the melanoma cell line suggests the arrest at the G_2_ phase. Furthermore, α-santalol inhibited microtubule polymerization in melanoma cells, indicating that the anti-mitotic activities of this sesquiterpene rely on its interaction with the microtubule network [122]. A third study by Santha et al. elucidated the effects of α-santalol on MCF-7 and MDA-MB-231 breast cancer cell lines and reported that it causes a G_2_/M-phase arrest in both cell lines, which was associated with the decrease of cyclin A [123]. However, the authors did not investigate further the anti-mitotic mechanism.

### 4.11. Xanthatin

Xanthatin is a xanthanolide sesquiterpene lactone isolated from plants of the *Xanthanium* spp. (Asteraceae family). This compound does not have a 5-membered ring but contains an α-methylene-γ-lactone (Table 3). Treatment of A549 non-small-cell lung cancer cells with 40 µM xanthatin for 24 h induced accumulation of cells at the G_2_/M-phase. The arrest correlated with a dose-dependent reduction of Chk1, Chk2, and an increase in phosphorylation of Cdk1, suggesting a G_2_ arrest, followed by apoptosis [124]. The authors did not provide a description of the target or the molecular pathway that was affected by xanthatin.

### 4.12. Zerumbone

Zerumbone (Table 3) is a sesquiterpene isolated from the essential volatile oil of rhizomes from the edible wild ginger *Zingiber zerumbet* (Zingiberaceae family) [125]. It was reported that zerumbone does not affect normal human peripheral blood mononuclear cells, but is cytotoxic to Jurkat leukemia cells. It arrests treated cells in the G_2_/M-phase of the cell cycle in a time (24, 48, and 72 h) and concentration (26, 42, and 57 µM) dependent manner, followed by apoptosis [125]. Chan et al. found that 30 µM zerumbone had anti-proliferative activity to PC-3 and DU-145 prostate cancer cell lines inducing a G_2_/M-phase arrest at 8 h, and increased the levels of PH3, cyclin B, and MPM2 expression, a mitotic marker [126]. It was shown that zerumbone caused a mitotic arrest by targeting tubulin/microtubules and disrupting microtubule dynamics, which led to the formation of aberrant monopolar and multipolar spindles [126]. The authors described other effects upon cells treated with zerumbone, which included endoplasmic reticulum stress and mitochondria-mediated apoptosis. In addition to caspase-dependent apoptosis, zerumbone also induced autophagic cell death mediated by a decrease of light chain 3 (LC3), a marker for autophagy [126,127]. Xian et al. reported that a 24 h treatment of NB4 leukemia cells with 10 µM zerumbone arrested them in the G_2_ phase of the cell cycle, as characterized by phosphorylation of Cdk1 on Tyr15. This arrest was followed by apoptosis, due to loss of the mitochondrial membrane potential [128]. When tested on Caov-3 ovarian cancer cells, zerumbone induced apoptosis and cycle arrest at G_2_/M-phase in a concentration--dependent manner (from 4 to 45 µM) [129].

## 5. Conclusions

**Figure 2:**
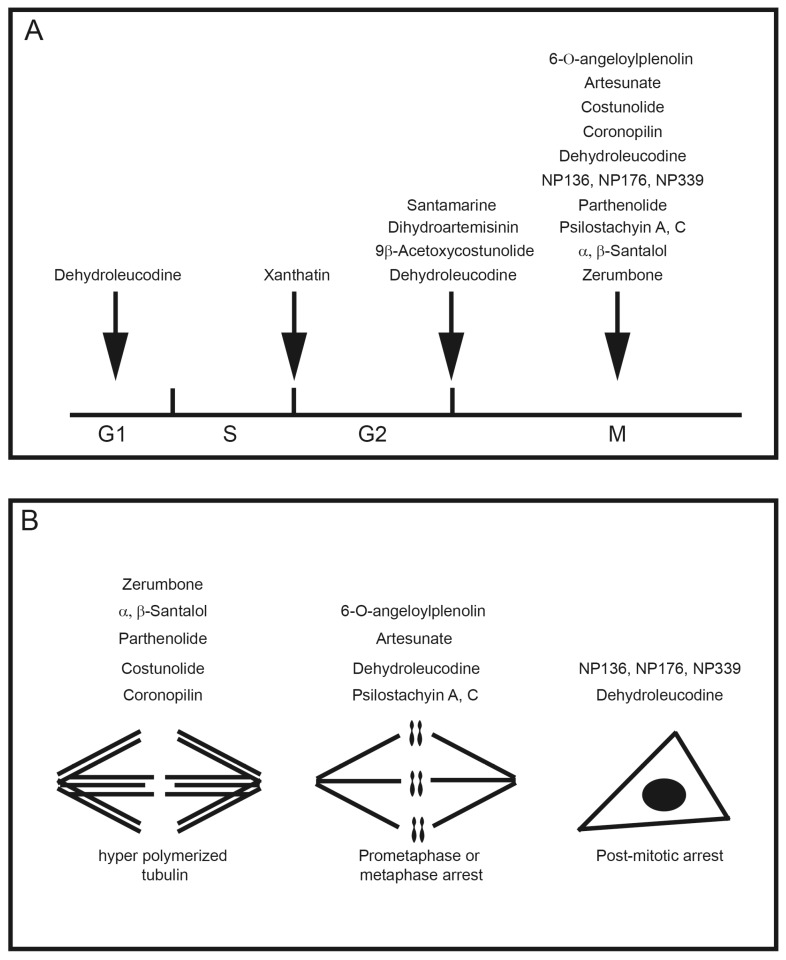
(**A**) The position of the cell cycle arrest relative to the phases of the cell cycle are given for the compounds. (**B**) The types of mitotic and post mitotic arrests are given for the compounds.

In the review of sesquiterpene molecules that affect human cells in assays in vitro, we highlighted those for which the data clearly demonstrated an arrest in the M-phase of the cell cycle. These data include evidence of molecular or cellular events such as phosphorylation of histone H3, condensed chromosomes, or a mitotic spindle (microtubules). These data could be supplemented with other data such as elevated levels of cyclin B, and non-Tyr15 phosphorylated Cdk1, which would be present in mitotic cells, but singly would be insufficient to determine if a cell is in mitosis or in G_2_ phase. In some publications, measurements of 4N DNA amounts by flow cytometry that indicated a cell cycle arrest; however, without additional data, it was not possible to know if the arrest was in M-phase. The sesquiterpenes that arrest cells in mitosis include 6-OAP, artesunate, coronopilin, costunolide, NP136, NP176, NP339, parthenolide, psilostachyins A and C, α- and β-santalols, and zerumbone (Figure 2). A second group of sesquisterpenes caused G_1_ or G_2_-phase arrests. This group includes 9β-acetoxycostunolide, dihydroartemisinin, dehydroleucodine, santamarine, and xanthatin.

The biomolecules that were targeted by the sesquiterpenes include components that regulate the mitotic spindle or interact with tubulin directly, which caused changes in tubulin stability, or microtubule hyperpolymerization or cytokinesis defects (Figure 2). A second activity that caused a mitotic arrest was linked to inhibition of ubiquitin-proteasome pathway. In the majority of cases, the pathway leading to a deformed spindle was not identified. The precise mechanism of how sesquiterpenes cause a mitotic arrest is not known, although there is a consensus that it might be linked to the metaphase-anaphase transition [102,107,109,120,130]. As described by Liu et al, in their analysis of 6-OAP, these phenotypes could be caused by inhibition of regulatory steps required for proteolytic pathways [102].

A structural comparison of the compounds that induce a mitotic arrest produces a complex picture because both sesquiterpene lactones and non-lactone sesquiterpenes are present. The group comprised of sesquiterpene lactones includes: 6-OAP, coronopilin, costunolide, 9β-acetoxycostunolide, dehydroleucodine, NP136, NP176, NP339, parthenolide, psilostachyins A and C, santamarine, and xanthatin. The second group includes artesunate and dihydroartemisinin, the α- and β-santalols, and zerumbone. It is hypothesized that the α,β-unsaturated carbonyl group is responsible for the activity of sesquiterpenes, including the anti-mitotic activity and checkpoint abrogation activity [107,110,115,120,130]. The hypothesis is supported by the demonstration that modification of the α,β-unsaturated carbonyl group by reaction with β-mercaptoethanol renders sesquiterpenes inactive in mitotic and checkpoint assays [120]. These data are further supported by mechanism of action studies in non-mitotic processes in which cysteines are covalently modified by a Michael addition reaction, rendering the polypeptide inactive.

**Figure 3:**
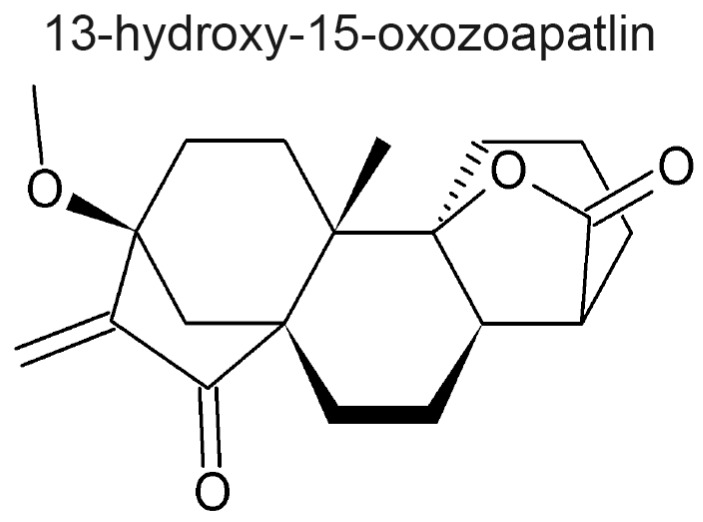
Structure of the *ent*-kaurane, 13-hydroxy-15-oxozoapatlin. This natural product has an α,β-unsaturated carbonyl, and an anti-mitotic activity similar to some of the sesquiterpene lactones reviewed here.

Another compound, 13-hydroxy-15-oxozoapatlin (OZ), with a similar structure to sesquiterpene lactones, exhibits similar anti-mitotic activities [130] (Figure 3). This natural product is not a member of the sesquiterpene lactone class because it is an *ent*-kaurane diterpenoid. OZ was isolated from the bark of the South African tree *Parinari curatellifolia* from the Chrysobalanaceae family. Although OZ has a core structure different from the sesquiterpene lactones reviewed here, it has an α,β-unsaturated carbonyl group that makes it reactive to nucleophiles and it has anti-mitotic properties upon treated cells. OZ treated cells exhibited atypical, disorganized mitotic spindles, although microtubule polymerization or depolymerization were not affected when tested in a purified system in vitro. Furthermore, OZ was rendered inactive after reaction with β-mercaptoethanol in a DNA damage checkpoint assay [130]. These data further support the notion that the lactone plays a key role in the mitotic arrest as well as other biological activities of these natural products.

Knowing that 90% of polypeptides in human cells have cysteines, one could assume that sesquiterpenes could react with nearly any protein. The presumed mechanism of action of generic cysteine modification is at odds with the specificity required to arrest a human cell in mitosis. A cell that enters mitosis requires the exquisite coordination of thousands of active proteins, including those involved in the most fundamental processes such as ribosome function to synthesize the cyclin required to initiate Cdk1 activity, and ATP synthesis for metabolism and phosphorylation events that characterize mitosis (such as phospho-histone H3). Yet the mitotic arrest phenotype described in the literature and observed by our laboratory indicates that a non-specific cysteine modification of proteins is insufficient to explain the action of sesquiterpenes. We predict that specific protein targets are inhibited by a subset of sesquiterpene molecules and this protein(s) is required for a mid to late event in mitosis, such as the metaphase-to-anaphase transition. Among the presumed targets of sesquiterpenes, tubulin and protein degradation pathways have been considered. Although these targets may not be exclusive, they would need to account for one observation common to all mitotic arrest phenotypes observed by sesquiterpene inhibition, the percentage of cells arrested in mitosis never arrives to near 100% as is the case with tubulin poisons such as nocodazole [131].

We propose that some of the chemical biology of sesquiterpenes be reinvestigated. In our analysis of the literature, the most common biological activity was that of cytotoxicity, which would be expected from non-specific Michael-type additions. Tests in our laboratory led us to the observation that a subset of sesquiterpene molecules inhibit mitosis, and this is a step that occurs before cell death [132,133]. It is possible that the number of compounds with anti-mitotic activity amongst the 5000 described sesquiterpene molecules is underestimated. We predict that as natural compounds, some sesquiterpene molecules may have invaluable medicinal properties through their cell cycle arrest activities. Furthermore, investigation of the chemical biology of sesquiterpene lactones opens the possibility of gaining further insight into the process of mitosis.

## Figures and Tables

**Table 1:** The names of plant taxonomical families that are known producers of sesquiterpene molecules. The common names and references are also provided.

Plant Family	Common Name	Reference
Acanthaceae	Acanthus family	[24]
Anacardiaceae	Cashew family	[11]
Apiaceae	Celery family	[24]
Araceae	Aroids family	[5,48]
Asteraceae	Sunflower family	[5,10]
Cactaceae	Cactus family	[5,48]
Euphorbiaceae	Spurge family	[5,48]
Lauraceae	Laurel family	[24]
Magnoliaceae	Magnolia family	[24]
Menispermaceae	Moonseed family	[24]
Rutaceae	Citrus family	[11]
Solanaceae	Nightshades family	[5,48]
Winteraceae	Winter’s Bark family	[24]

**Table 2:** Sesquiterpene lactones that are in clinical trials as anti-cancer drugs. The table shows the structures of the three compounds (artesunate, LC-1, and L12ADT), the sources, the mechanisms of action and references.

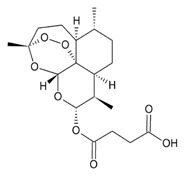	**Artesunate**
	Semi-synthetic derivative of artemisinin. Targets the iron group content by catalyzing the generation of free radicals from the bridged endoperoxide group. Phase II clinical trials for cervical intraepithelial neoplasia, colorectal cancer, non-small cell lung cancer, metastatic uveal melanoma, and laryngeal squamous cell carcinoma. References [87,88,89,90,91,92].
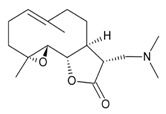	**Dimethylaminoparthenolide (LC-1)**
	Oral bioavailable analogue of parthenolide. Inhibition of NF-κB DNA binding and activation of p53 protein. Phase I clinical trials against acute myeloid leukemia (AML), acute lymphoblastic leukemia (ALL) and other blood and lymph node cancers. References [72,87,97,98].
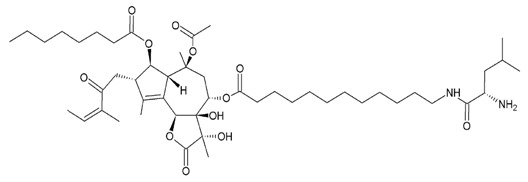 **L12ADT (8-*O*-(12-{l-leucinoylamino}dodecanoyl)-8-*O*-debutanoyl-thapsigargin)**	
Semi-synthetic derivative of thapsigargin. Inhibitions protein synthesis and sarco/endoplasmatic reticulum (ER) and ATPase (SERCA). Phase I clinical trials for refractory, advanced or metastatic solid tumours, and phase II clinical trials for glioblastoma. References [93,94,95,96].	

**Table 3:** The names and structures of sesquiterpenes that have cell cycle arrest or mitotic activity are given.

Compound	Anti-Mitotic Activity	Reference(s)
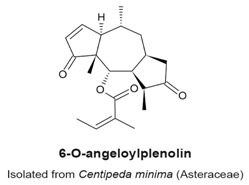	Prometaphase arrest, activation of the Cdk1 dimer and increase of PH3 levels. Activation of the spindle assembly checkpoint, failed activation of APC/C and decrease of ubiquitinated cyclin B levels. Binding at the Skp1-Skp2 interface and inhibition of the SCF-NIPA complex.	[100,102,103]
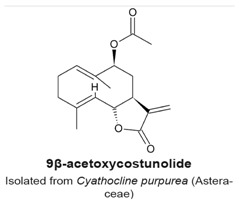	G_2_/M phase arrest (not specified if it is a G_2_ or an M arrest).	[104]
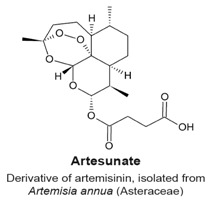	G_2_/M phase arrest. Presence of multiple centrosomes, multiple spindle poles and multinucleated cells. Cell cycle arrest caused by a defect in cytokinesis.	[105,106]
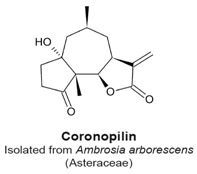	Mitotic arrest. Sustained levels of cyclin B and PH3, suggesting metaphase arrest. Covalent interaction with tubulin nucleophilic groups, causing hyperpolymerization of tubulin.	[107]
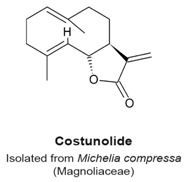	Inhibition of tubulin carboxypeptidase activity and restoration of normal levels of Glu-tubulin. Mitotic arrest followed by increased phosphorylation of PH3. Metaphase arrest and formation of short and dense microtubule fibers.	[108,109,110,112]
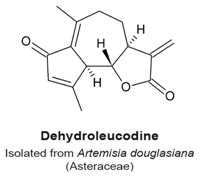	Delay in mitotic entry and an increased duration of the mitotic phase. Upregulation of PH3. Temporary mitotic arrest and final accumulation of cells in a G_1_ phase senescence.	[113]
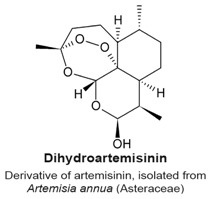	G_2_/M phase arrest (not specified if it is a G_2_ or an M phase arrest). Increased levels of cyclin B and decreased levels of Wee1.	[106]
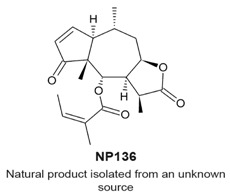	Underduplication of centrioles. Formation of monopolar spindles. Aberrant chromosome segregation.	[114]
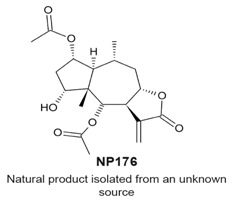	Underduplication of centrioles. Formation of monopolar spindles. Aberrant chromosome segregation.	[114]
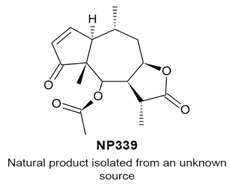	Underduplication of centrioles, formation of monopolar spindles, aberrant chromosome segregation. Does not affect a non-transformed cell line. Impairs centriole formation by modulating NF-κB signaling.	[114]
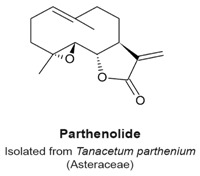	M phase arrest. Inhibition of tubulin carboxypeptidase. Decrease of the pool of detyrosinated tubulin and stabilization of microtubules. Reduction of microtentacle formation and tumour cell attachment.	[108]
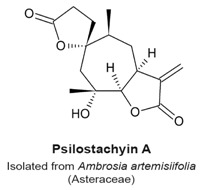	Prometaphase-like arrest. Condensed chromosomes not properly aligned. Polymerization in vitro of purified tubulin was not affected. Disorganized mitotic spindles. Mercaptoethanol-psilostachyin A does not cause a prometaphase-like arrest.	[120]
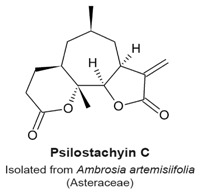	Mitotic arrest at a prometaphase-like stage. Condensed chromosomes not properly aligned. In vitro polymerization of purified tubulin was not affected. Disorganized mitotic spindles.	[120]
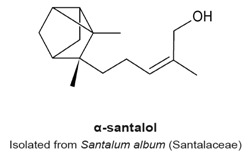	G2/M phase arrest. Formation of aberrant mitotic spindles (punctate, multipolar or monopolar). Decreased polymerization of purified tubulin in vitro. Binding to the colchicine site on tubulin.	[121,122,123]
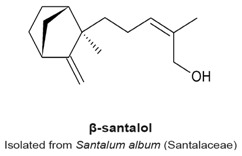	G_2_/M phase arrest. Formation of aberrant mitotic spindles (punctate, multipolar or monopolar). Decreased polymerization of purified tubulin in vitro. Binding to the colchicine site on tubulin.	[121]
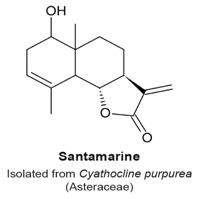	G_2_/M phase arrest (not specified if it is a G_2_ or an M phase arrest).	[104]
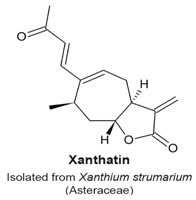	Accumulation of cells in G_2_ phase. Phosphorylation of Cdk1.	[124]
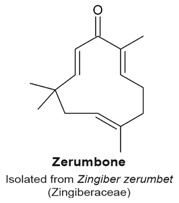	Mitotic arrest characterized by increased levels of PH3, cyclin B1, MPM2 expression. Disruption of microtubule dynamics and formation of aberrant monopolar and multipolar spindles.	[125,126,128,129]
